# Inflammation, Ulceration and Induration of the Os and Cervix Uteri, and Use of the Speculum

**Published:** 1858-09

**Authors:** W. M. Chambers

**Affiliations:** Charleston


ARTICLE V.

### INFLAMMATION, ULCERATION AND INDURATION OF THE OS AND CERVIX UTERI, AND USE OF THE SPECULUM.

BY W. M. CHAMBERS, M. D., OF CHARLESTON.

(Read before the Esculapian Society, at its Annual Session in October, 1857.)

The facts and views of the following gentlemen will be made use of indiscriminately, viz: Drs. Lee, Bennett, West, Whitehead, Tyler Smith and Churchill, of London; Dr. Simpson, of Edinburgh; and Drs. Meigs and Miller, of our own country. These, I believe, comprise the names of all who have figured extensively in the investigation of the questions under consideration.

Claiming to have been somewhat of an observer of the progress of medical science for the last twenty years, I have no hesitation in saying that more light has been thrown upon the diseases and natural functions of the female reproductive organs, than upon either of the other two arbitrary divisions of professional knowledge. It is impossible to state which of two causes have contributed most to this very desirable state of things. Whether the fact, that it has laid claim to a higher position than that formerly assigned it, of being consigned to old granny women of both sexes, or by that of having the attention of men of superior minds directed to its consideration. Although great improvement has taken place in surgery and practical medicine, yet not to the extent which has been witnessed in this department of our profession.

The attention of medical men of distinction was more particularly directed to this department about 1845 or '46, since which time the anatomy, physiology, pathology, diagnosis, symptoms and treatment have undergone an ordeal which is highly creditable to many of those whose names we have mentioned. At the same time, much has been said in an angry, snarling way, which is a source of regret in more respects than one. One source of regret is, that in this state of things the statements of the parties are so positive, and yet so divergent, that it makes it no easy thing for those who are not familiar with the subject, to arrive at any very satisfactory conclusions as to who is right or who is wrong.

I really feel at a great loss how to present the subject, and approach it with fear and trembling. It is that kind of trembling a man feels when he takes hold of a knife for the first time, to perform an important operation. The reason I tremble is not so much from want of familiarity with it, as because I am not yet satisfied what shape to put it in so as to render it most acceptable to the society, but believe, to eschew system, and present the matter as it occurs to my mind, will be attended with as much success as in any other form it could be presented.

Ever since I have been a practitioner of medicine, a class of cases have presented for treatment with about the following symptoms. In most instances the patients are married women, some of them have been married for years and never been pregnant; another class have been frequently impregnated, but never carried the fœtus to the full term of gestation; while yet another class have had one or more full term healthy children, and are now either barren or frequently aborting or miscarrying. I do no not say that virgins are never afflicted with the symptoms about to be enumerated, but, so far as my own observation extends, the instances are exceedingly rare. In most cases, you will elicit about the following account of the condition of the patient: feebleness is written on her brow and seen in her very motion. She is generally thin and pale, or else subject to an occasional flush of the face. The tongue is broad and flabby.

The pulse is quick and sometimes intermitting. Upon inquiry, it will be found that she has lost from ten to thirty pounds of flesh. She will tell you she has pain in her back, pain in her side, pain in her head—the recumbent posture affords little or no relief to pain in the back. She has palpitation, which may be produced by slight exertion, or may awake her in the night or while she is in a perfect state of repose. She has tenderness over the region of the stomach; tenderness somewhere in the course of the spinal column; sour stomach, which is indicated by acid eructations; costiveness and diarrhœa alternately, neither condition evincing any want of biliary secretion; the appetite is variable, but in most instances is poor; she is easily frightened, and nervous to an intense degree. She has leucorrhœa in variable forms, worse or more profuse after the regular monthly discharge, which by the way may not be so very regular, either too often or not often enough, or too profuse or spare, sometimes continuing eight or ten days or only one or two. The leucorrhœa may be of a milky consistence, or thick and resembling the white of an egg, or yellow, or green, and smelling very offensive. After each monthly evacuation, when there is leucorrhœa following, you will be told that there is great pain in voiding urine, with a frequent desire to do so. In some few instances there may be no leucorrhœa, with, however, all the other symptoms. The patient is not confined to the bed all the time, but has to take the recumbent posture frequently through the day. These symptoms have been present in a greater or less degree for months or years. It will be found, on examination, that the heart presents no organic lesion as far as auscultation will reveal anything. The most searching investigation will not detect any lesion of the stomach, liver or bowels. There are no lung symptoms, and so far as the brain is concerned, none among us would be rash enough to pronounce it in an abnormal condition, unless through the medium of sympathy. The skin and kidneys are performing their functions in a blameless way. Now it used to be the case that, finding these symptoms with the normal conditions we have spoken of, the physician was in the habit of saying to his patient, "Well, madam, I think you may be relieved," and has made, perhaps, a promise of a cure.

He starts out to relieve some of the more prominent functional disturbances, and at first for a few days he is in raptures, so is his patient, but weeks and months roll on and still these symptoms will occur again and again. Such cases, before he is long in the profession, become the plague of his life. His books are searched in vain, his brain is racked, his nights are sleepless, and his reputation is on the wane.

But let us see what he has been doing. He has cupped the spine, and made counter irritation; he has cupped the sacrum, and blistered it, and kept it sore; he has given mercury and alteratives of various kinds; given tonics; ordered exercise to be taken without fatigue, and a diet to suit these conditions; used injections for the vagina, and kept them in constant use, and yet neither the general nor local symptoms are relieved, and this course of practice is precisely in keeping with that recommended by Dewees, Churchill, Lee, Meigs, Ashwell and Columbat, and more recently by West and Tyler Smith. I would not say, in any instance, that the course recommended by those gentlemen would be unsuccessful, for there may be cases with well marked symptoms where there is great recuperative power, when a cure might possibly be effected. The treatment here spoken of corresponded with the notions entertained by writers upon female diseases, and was in accordance with their views of the pathology of the reproductive organs, with this exception that we were told that in some of the cases we would find prolapsus of the uterus, and for this various kinds of pessaries were devised, and in some instances we were told to cut out a longitudinal fold of the mucous membrane lining the vagina, and thus narrow its walls and prevent the descent of the womb in that manner. The profession sat down and folded their hands with this state of things, and left their patients to eke out miserable existences.

The publication of Dr. Bennett's papers in the London *Lancet*, and the reading of his paper to the College of Physicians in the year 1845, startled the medical men of London like a shock of electricity. He said these symptoms were caused in a great degree by inflammation, ulceration or induration of the os or cervix uteri, or both, and that the constitutional symptoms would not yield until the local disease was removed, which could alone be accomplished by local remedies. I did not see Dr. Bennett's views of this subject until I had read Dr. Whitehead's work on Abortion and Sterility, in which he sustains the views of Dr. B., and in a very clear and concise manner gives cases to illustrate his views. Since that time I have made myself somewhat familiar with the views of Dr. B. Dr. Robert Lee, for whose talents I entertain the highest regard, read a paper upon this subject before the Medico-Chirurgical Society shortly after Dr. B.'s publication, in which he uses the following emphatic language, which may be found in the transactions of the society in vol. XXVIII. page 275, "*Neither in the living nor the dead body* have I ever seen ulceration of the os and cervix except of a *specific* character, and especially scrofulous and cancerous." Now if it be true that no other ulcerations ever occur but from cancer and scrofula, then Dr. B.'s notions and views amount to no more than a myth. In 1850, Dr. Tyler Smith read a paper before the same society, I believe, which was subsequently published, in which he elucidates similar views to those of Dr. Lee. In 1854, Dr. Charles West delivered the Croonian lectures. His subject was "An Inquiry into the Pathological Importance of Ulceration of the Os Uteri," in which he admits the existence and the *frequent* existence of inflammation, ulceration and induration of the os and cervix uteri, but concludes their existence are of slight pathological importance, and "of small semeiological value; a casual concomitant, perhaps, of many disorders of the womb, but of itself giving rise to few symptoms, and rarely calling for special treatment." In the October number of the *Medico-Chirurgical Review* for 1854, at page 248, Dr. Churchill reviews the lectures of Dr. West, and coincides with his views to a very great extent, but thinks he admits the existence of ulceration too much. In 1855, Dr. Tyler Smith publishes his great work on leucorrhœa, and there virtually admits nearly all Dr. Bennett has contended for in the way of pathology, but condemns the use of the stronger caustics and the actual cautery. Dr. Bennett's doctrine may be presented in a few words. He believes that inflammation, ulceration or induration of the os and cervix uteri may be regarded as the first in a train of processes, which are the direct or indirect occasion of by far the greater number of ailments of the generative system. Dr. Simpson, of Edinburgh, bears ample testimony both as to the pathology and treatment of Dr. Bennett, as do Drs. Meigs and Miller of our own country. Thus it will be perceived that there is a strong array of talent on both sides.

A number of fastidious gentlemen have objected to the course of Dr. Bennett, on the ground that it is an unnecessary exposure of females. If it is true, however, that they can be benefited by exposure and in no other way, then the objection is worthless.

Let us now review these gentlemen. Dr. Bennett reads his paper and subsequently publishes his views, in which he contends for the principles already stated, viz: that ulceration of the cervix produces a train of symptoms which will not always yield to general treatment, and cannot be accounted for upon any principle of rationality unless it is found in the os or cervix, and cannot be cured except by local applications. Dr. Lee and Dr. Tyler Smith read papers in which they deny the existence of ulceration of the os, but most strangely admit (both of them) that they frequently found granulations secreting a peculiar pus or something of the kind, and fissures and indurations of the lips of the os, and sometimes abrasions, none of which were preceded by inflammation or attended with ulceration. Dr. West subsequently read his lectures, in which he confessed the frequency of both inflammation and ulceration of the os and cervix, but denies their importance. Dr. Churchill reviews and says that abrasions have not existed, but instead it was congestion, thus admitting a state of inflammation to follow. Dr. Tyler Smith follows with his work on leucorrhœa, in which he contends for the treatment of the granulations, indurations, etc., by the same class of remedies recommended by Dr. Bennett. Dr. Bennett in the May number of the London *Lancet* for 1856, page 397, thus concisely states his position: "Now if I, as a practitioner, have found, during a long series of years, that I have constantly been consulted by young sterile married women, in whose history I can trace the evidence of uterine mischief, dating from the earliest period of their married life, or even from an epoch antecedent to it; if, on examination, I find some chronic inflammatory uterine lesions, say ulceration; if I treat the local disease and cure it; and if a considerable number of these women subsequently became fertile,—am I not warranted in considering the local disease as the cause of their sterility? If, again, I find married women who have had children often becoming sterile for years, after a tedious or instrumental labor, which has left traces of uterine suffering; if, discovering this condition to be connected with local inflammatory mischief, I remove it by treatment, and they subsequently in very many instances again become pregnant,—am I not warranted in considering the temporary sterility of these women as occasioned by the temporary local disease? If, on the other hand, I find that women who are continually aborting or miscarrying are generally suffering from symptoms of uterine ailment, and present, on examination, local inflammatory lesions, mostly inflammatory ulceration, and on thoroughly removing these lesions, I find that a large proportion of them at once go to the full time, and are delivered of live children,—am I not warranted in concluding, that in these females the existence of inflammatory disease was the cause of the abortions and of the premature termination of the pregnancies." In this late review of the present state of uterine pathology, Dr. B. reports cases by the hundred, of complaining women whose symptoms would not yield to ordinary remedies, but readily to local remedies and appliances in conjunction with general treatment. Dr. Whitehead gives, as before remarked, the cases as he goes along, and fairly coroborates Dr. B. Dr. Simpson goes still farther than Dr. B. in the application of caustics, and endorses his views throughout. Dr. Meigs relies more on general remedies, but says, "where there is albuminous leucorrhœa it is a sign of inflammation of the cervix, in which is included the canal with its copious muciparous apparatus. It is as much a surgical disease as an ulcer of the leg, as anthrax or conjunctivitis. When the surgical disorder is cured the sign disappears," but he denies the ulceration, and, contrary to Dr. Tyler Smith, admits the inflammation. Dr. Henry Miller, of Louisville, Ky., supports the views of Dr. Bennett, and to my own knowledge has been wonderfully successful in curing cases of long standing which had failed to yield to the old plan of treatment in the hands of the most skillful men in the profession. This is bringing the matter pretty near home, and if I may be allowed I will add that since 1849 I have been in the habit of serving a number of the cases whose symptoms I have given some pages back, and the success has been of the character spoken of by Drs. Bennett, Simpson and Whitehead.

I confess that I have never resorted to the more formidable caustics and escharotics, I having probably felt my way with more caution than some others, and in the majority of instances made an effort to benefit the patient by some constitutional treatment, and at all events improved the digestive powers before local treatment was resorted to; but at the same time I think I can see a propriety in the use of escharotics, especially in indurations and hypertrophies.

I should feel that my paper would be incomplete unless reference was made to the local pathology of the disease. When it is found necessary to make an examination (and just here we might, as I conceive, argue the propriety of when that should be done), I contend that a digital scrutiny should be made whenever there is a strong suspicion of uterine derangement (and there are very few who are on the list of complaining women who are not thus deranged); and if, instead of the smooth, round, greasy feel which a healthy condition would indicate, we find a widening of the os, or tenderness, or displacement, or hardness, or roughness, it will be the imperative duty of the practitioner to resort to the speculum. The utmost concern for the welfare of the patient should be manifested, and when the speculum is used he will find revealed a state of things which, to those unaccustomed to the use of this instrument, will be surprising, a broad surface of granulations spread out around the os extending into the cervix. Sometimes this is so extensive that the entire surface is not perceptible through an ordinary glass or ivory speculum; again it may be that the lips of the os are only parted, and the granulations are entirely within the cervix. Fissures sometimes present themselves, and a thickened hard edge of the os, either of the anterior or posterior lip, or both, are perceptible. In a number of instances, a plain ulcer with well defined edges may be seen presenting on the edge of the os, or on an out-turned lip, from cervical granulations. In some instances there are granulations, and in some it is a smooth, polished, raw surface, with many variations and modifications.

Now Dr. West says these conditions have nothing to do with the production of general symptoms. I would suggest that the opposite sex are not addicted to these long spells of unaccountable sickness. That an increased discharge, whether healthy or unhealthy, will produce debility—weakness; that no part of the organism is so destitute of vitality but, if it is kept constantly irritated, must produce more or less debility of the general system; that it is not conclusively settled whether or not the cervix is less vitalized than other organs or textures; and if it were settled that it was, it would not prove conclusively to my mind that the drain upon the system which is witnessed in the discharge would not drag down the entire organism, and produce the very train of symptoms which has been enumerated, nor that the arresting of this drain, and curing these granulations, and ulcers, and indurations, would not be attended with benefit. I say it would not convince me that Dr. West's views are correct, because I have the testimony of others as reliable as he is, that the pathological condition exists (he admits this much himself), that the train of symptoms are present, that they will not yield to general remedies, that they will to local remedies, that so soon as the local mischief is removed the patient gets well; and besides this testimony, I have my own observation, which corroborates all these points. In order that I may not over or under estimate the number of cases which I have treated, both in conjunction with others and alone, I have taken the trouble to count the patients who I have known to be treated by local remedies and find that they amount to fifty, and I can safely say that a cure was effected in nearly every instance. In two instances the relief was not perfect, but in one the patient had gained strength and became cheerful, and the remnant of the ulcer was scarcely perceptible, and I have not a doubt but the cure would have been perfect if she had remained under treatment, but her husband packed up his plunder and on a night left for parts then unknown to me. The other was a lady, forty-two years old, barren, and, I fear, too highly endowed with sexual propensities to be true to her lord and master, and I am not sure whether she recovered or not, but the last time I heard from her was some six months after my attention ceased, she had not become pregnant to her knowledge. Thus it will be seen that there are really forty well marked recoveries, which is enough, in my estimation, to establish not only the propriety but the necessity of local applications.

From these facts, gentlemen, from others and my own observation, it is plain that I should resort to local applications whenever the symptoms would resist general treatment, but at the same time I heartily detest and loathe the man who would needlessly subject a woman to either digital or instrumental examination. Having satisfied my own mind upon this subject, I shall at once proceed to the local treatment. Before doing so, however, I will add, that I do not think in any instance the importance of the disease should be magnified in order to induce a woman to submit to the examination.

After the digital examination is made, which may be done while the woman is in the recumbent posture (either on her back or side), or in the erect posture (and it will be frequently found that this latter is necessary, in order to make out the condition of parts, as, for instance, when there is prolapsus or other displacement), the patient will then be told that there is a disease at the neck of the womb, and that she will have to undergo (not submit) further examination, and that she may have some lady friend present on a certain day and hour, and be prompt to the minute. He will select a light in the second floor, if possible, placing the woman with her hips to the light; but before he commences, he should have a bowl of water, some sweet oil or sweet lard, a ball of soap, a towel and a sheet. The light should enter the room from only one source, if possible, and that fronting the side of the bed, and for a steady light I should select the hour of two p. m., and let it come from the north. It is best the patient should lie on a mattress or straw bed, in which case it will not be necessary to have her hips to the edge of the bed, otherwise it might be; nor would it be of the first importance then to have the side of the bed to the light, but if it is just as convenient it is best. It is a matter of habit with the practitioner whether the patient is placed on her back or on the left side. I prefer the latter, but have practiced both. After the patient is thus placed, the sheet should be folded once only, and taking it in the hand, the physician walks to the bed, conversing upon some trivial subject in which the patient is interested; he places the sheet under her clothes and next to her skin, bringing the ends of the sheet around her legs, and places the corners against the external organs. He is then to make a digital examination and find out the condition and position of the os, and then after oiling his instrument (the valvular speculum, and I give preference to the form valve), and having his probang well armed with a soft sponge, to lay on the bed and within reach, he continues the conversation, foreign from the one he is engaged in, and introduces the speculum, which is accomplished by taking the instrument in his hand or thumb and fingers, placing the end within the vulva and pressing gently upwards and backwards, having the anatomical structure of the parts in view, the handles pointing to the sacrum. After it has reached the point in the vulva, where the blades are fastened, he should cautiously and steadily approximate the blades, which will at once enable him to withdraw the director, when with the screw he brings the blades together, keeping in view all the time the necessity of grasping the instrument in his left hand with folds of the corners of the sheet around it, so as to prevent exposure of external parts. Thus he has brought all the internal organs into full view. His attention must be directed first to the condition of the os and cervix, and he may compare the membrane lining them with the color of the membrane which lines the mouth and fauces of his patient, which he will have done before he commences the investigation which is now going on. If there be much secretion of pus or other material, he can remove it with the probang, and then determine the nature of the case. If there be fissures, granulations or ulcers, he can pretty well determine their extent, and cautiously withdraw his instrument, and at once introduce the ivory or glass speculum; and here is a matter of the utmost difficulty with beginners—

no force must be attempted upon any pretext whatever. After passing the vulva, the great object is to have the neck of the womb fill the end of the speculum. If he has well studied the position of the os, he will commonly succeed the first effort; if not, he may have to partially withdraw it a number of times, but as a general thing he will succeed by holding the outer end of the instrument as high up toward the symphysis pubis as is compatible with the comfort of his patient, whom he is to inform that it is not necessary to hurt her, and if he should to let him know. Some little manipulation will be found necessary in all cases, and the physician must not be discouraged if he has to spend some time in finding the os. Once found, with this instrument he is to have in readiness besides his probang, a caustic holder, armed with a smooth-ended piece of solid nitrate of silver and a very strong solution of the same, say forty to sixty grains to the ounce of rose water or rain water, and now exercising his judgment as to the intensity and extent of the disease, he applies to the full extent of the diseased structure. If he finds, instead of ulceration, granulation or fissures, a high grade of inflammation of either os or cervix, or both of these with the vagina, or the vagina alone, he will best succeed by the application of leeches, which may be followed by injections of nitrate of silver in solution, from six to twelve grains to the ounce of water, repeated every day. If it is of the class of cases where the nitrate of silver in substance is used, the investigation and reapplication must be repeated every five or six days, until the patient is well, or it is found that the acid nitrate of mercury has to be resorted to, which is to be accomplished by the use of a piece of lint or wool tied on to a piece of whalebone, having the internal part of the syringe well oiled and the solution well squeezed out of the lint so that it be not too wet, and then before removing the speculum it should be so turned that any part of the excess of the acid might escape into a basin which should be at hand for that purpose. The reapplication of this substance should not be made in less than seven or eight days, as the eschar formed by it does not fall off before that time, and then I have rarely seen it applied more than once; indeed, the necessity for it did not exist, the nitrate of silver being generally sufficient to accomplish the remaining part of the cure.

It will thus be seen that I have not resorted to the potassa fusa nor the Vienna paste, nor actual cautery, having thus far succeeded without them.

I wish to add, before I conclude, that I am confident from my own observation that a great many of the cases of prolapsus and other displacements of the uterus owe their origin to ulceration of the os and cervix; and it doubtless occurs in the following manner: there is necessarily a determination of fluids to the lower portion of the womb, which increases its weight and renders the inferior parts for support least able to withstand the pressure.

Nothing has been said as to the causes which produce the disease. They are so prolific that their enumeration with the modus operandi would take up much of the valuable time of the society needlessly. Metritis is a prolific cause, sexual intercourse another, etc.

Dr. West says that ulceration, granulation, etc., "are of small semiological value," rarely if ever calling for the interference of the surgeon. I am inclined to think that the very argument he bases this conclusion upon is the one with which Mr. Bennett can sustain himself. For instance: Dr. W. says that this is true, from the fact, that the neck has a spare distribution of nerves, and in a healthy condition enjoys a low state of vitality as a consequence of this want of nervous distribution. Now, is it not true, that this very state of things would go to prove that the "vis medicatrix naturæ" would also be deficient, and, as a consequence, whenever that portion became diseased, nature would be incapable of performing a cure under ordinary treatment directed to the general health? then, if there is ever a necessity for local applications to cure local diseases, this is surely the case.

